# Burnout in the intensive care unit: organizational determinants in focus

**DOI:** 10.62675/2965-2774.20260175-2

**Published:** 2026-09-17

**Authors:** Regis Goulart Rosa, Carol Hodgson, Cassiano Teixeira

**Affiliations:** 1 Hospital Moinhos de Vento Internal Medicine Department Porto Alegre RS Brazil Internal Medicine Department, Hospital Moinhos de Vento - Porto Alegre (RS), Brazil.; 2 Universidade Federal do Rio Grande do Sul Porto Alegre RS Brazil Postgraduate Program in Pneumological Sciences, Universidade Federal do Rio Grande do Sul - Porto Alegre (RS), Brazil.; 3 Monash University School of Public Health and Preventive Medicine Australian and New Zealand Intensive Care Research Centre Melbourne Australia Australian and New Zealand Intensive Care Research Centre, School of Public Health and Preventive Medicine, Monash University - Melbourne, Australia.; 4 The Alfred Hospital Department of Physiotherapy Melbourne VIC Australia Department of Physiotherapy, The Alfred Hospital - Melbourne, VIC, Australia.

The sustainability of the critical care workforce has become a central concern in contemporary health systems. Burnout is no longer a peripheral issue; it is increasingly recognized as a system-level signal of misalignment between workforce demands and organizational capacity. Nowhere is this tension more visible than in intensive care, where clinical complexity, time pressure, and exposure to suffering converge in a uniquely demanding work environment.^(1–3)^

In this issue of Critical Care Science, Rezende et al. provide timely and important data on burnout and life satisfaction among intensive care unit (ICU) physicians in Brazil, based on a nationwide probabilistic survey designed to reflect the heterogeneity of the country's workforce.^(4)^ Their findings are difficult to ignore: among 2,338 physicians contributing 3,112 ICU employment links, nearly 70% exhibited at least one altered burnout domain (emotional exhaustion, depersonalization, or personal accomplishment) and more than half reported low life satisfaction. Beyond prevalence, the study shows how physicians working in ICU experience well-being differently across roles and ICU environments. Burnout was less frequent among older physicians, but consistently associated with workload intensity, particularly schedule overload, and inversely associated with recovery-related behaviors such as sleep quality, physical activity, and social engagement. Low life satisfaction followed a similar pattern, with additional associations observed for commuting burden and work outside certified intensive care practice. What distinguishes this study is less the magnitude of burnout, already well described, than the granularity with which it situates physician well-being within the structure of ICU work. By modeling data at the physician-employment link level, the authors capture a defining feature of many health systems, particularly in middle-income settings: fragmented employment across multiple jobs.

The signal that emerges is consistent with prior literature. Organizational load, including schedule burden, workload intensity, and job fragmentation, has been repeatedly associated with burnout and reduced well-being.^(5–7)^ These dimensions are closely related to broader features of ICU work, such as chronic understaffing, long shifts, overtime, and high patient acuity, which collectively contribute to sustained workload pressure. Conversely, recovery-related behaviors such as sleep, physical activity, and social engagement have been linked to better outcomes.^(8,9)^ Still, interpretation requires restraint. This is a cross-sectional study, and associations cannot establish causality or directionality. Physicians who sleep better may be less burned out, but it is equally plausible that lower burnout enables better sleep. There is also a risk of misplacing responsibility. The association between recovery behaviors and better outcomes should not be interpreted as support for exclusively individual-level solutions. Organizational interventions are at least as important as individual strategies, as they address the structural conditions that shape clinician well-being.^(10)^ While individual approaches may offer incremental benefits, evidence suggests that organization-directed interventions tend to produce more consistent and durable effects.^(11,12)^ This distinction has practical implications for intervention design. Burnout is often framed as a problem of resilience or coping capacity. However, when prevalence approaches 70%, the problem is unlikely to reside primarily at the individual level. Instead, these findings are consistent with a systems-level explanation. Intensive care unit work is organized in ways that may systematically erode recovery, including prolonged schedules, multiple concurrent jobs, and commuting burden. These structural factors are increasingly recognized as key determinants of physician well-being.^(9,13)^ Moreover, emerging evidence suggests that organizational culture influences burnout not only directly, but also indirectly through mechanisms such as moral distress and work–life imbalance, reinforcing the central role of system-level conditions.^(14)^

From a research perspective, priorities are clear. First, longitudinal studies are needed to understand trajectories of burnout over time. The lower prevalence among older physicians observed here aligns with prior studies but remains poorly explained. This pattern may reflect a combination of survivor bias, in which physicians more vulnerable to burnout reduce their workload or leave practice, and adaptive mechanisms, including greater autonomy, more stable roles, and refined coping strategies developed over time. Cohort effects and generational differences in career expectations may also contribute. Second, interventional research should move beyond individual-level strategies. While coaching and mindfulness have modest effects, organizational interventions appear more impactful but remain underexplored. Priority candidates include improved staffing ratios, more flexible rostering, reduced shift burden, and the redesign of roles and tasks to decrease fragmentation and inefficiency. Evaluating such interventions will likely require cluster-based designs, including cluster-randomized or stepped-wedge trials, given their system-level nature and the need to minimize contamination. In addition, these studies will require the use of complex intervention frameworks,^(15)^ as such strategies typically involve multiple, interacting components implemented within dynamic healthcare systems. Third, more evidence from low- and middle-income settings is needed. Workforce structure, employment patterns, and resource constraints differ substantially from high-income contexts and likely shape burnout in distinct ways. Finally, measurement remains an issue. Heterogeneity in burnout definitions limits comparability across studies and may affect prevalence estimates. In addition, stigma and concerns about disclosure may lead to underreporting, further complicating interpretation.

In summary, the study of Rezende et al.^(4)^ provides robust national evidence that burnout and low life satisfaction are highly prevalent among ICU physicians and are patterned along organizational lines. The message is not that intensivists need to cope better. It is that ICU work, as currently organized, may be misaligned with sustainable practice. Recognizing burnout as a system signal rather than an individual failure is a necessary first step. Acting on that signal is the challenge that follows.
